# Characterization of Hypoxia Signature to Evaluate the Tumor Immune Microenvironment and Predict Prognosis in Glioma Groups

**DOI:** 10.3389/fonc.2020.00796

**Published:** 2020-05-15

**Authors:** Wanzun Lin, Shihong Wu, Xiaochuan Chen, Yuling Ye, Youliang Weng, Yuhui Pan, Zhangjie Chen, Long Chen, Xianxin Qiu, Sufang Qiu

**Affiliations:** ^1^Department of Radiation Oncology, Fujian Cancer Hospital & Fujian Medical University Cancer Hospital, Fuzhou, China; ^2^School of Clinical Medicine, Fujian Medical University, Fuzhou, China; ^3^Division of Neurocritical Care, Huashan Hospital, Fudan University, Shanghai, China; ^4^Department of Radiation Oncology, Shanghai Proton and Heavy Ion Center, Shanghai, China

**Keywords:** hypoxia, gene set enrichment analysis, tumor microenvironment, hypoxia risk model, glioma, immune response

## Abstract

Glioma groups, including lower-grade glioma (LGG) and glioblastoma multiforme (GBM), are the most common primary brain tumor. Malignant gliomas, especially glioblastomas, are associated with a dismal prognosis. Hypoxia is a driver of the malignant phenotype in glioma groups; it triggers a cascade of immunosuppressive processes and malignant cellular responses (tumor progression, anti-apoptosis, and resistance to chemoradiotherapy), which result in disease progression and poor prognosis. However, approaches to determine the extent of hypoxia in the tumor microenvironment are still unclear. Here, we downloaded 575 LGG patients and 354 GBM patients from Chinese Glioma Genome Atlas (GGGA), and 530 LGG patients and 167 GBM patients from The Cancer Genome Atlas (TCGA) with RNA sequence and clinicopathological data. We developed a hypoxia risk model to reflect the immune microenvironment in glioma and predict prognosis. High hypoxia risk score was associated with poor prognosis and indicated an immunosuppressive microenvironment. Hypoxia signature significantly correlated with clinical and molecular features and could serve as an independent prognostic factor for glioma patients. Moreover, Gene Set Enrichment Analysis showed that gene sets associated with the high-risk group were involved in carcinogenesis and immunosuppression signaling. In conclusion, we developed and validated a hypoxia risk model, which served as an independent prognostic indicator and reflected overall immune response intensity in the glioma microenvironment.

## Introduction

Hypoxia is a hallmark of the tumor microenvironment; growing tumors frequently exist in hypoxic conditions because of insufficient blood supply (1). Unlike healthy cells, tumors initiate a wide array of adaptive behaviors (e.g., angiogenesis, proliferation, and invasion) in response to low oxygen levels to ultimately promote a more aggressive tumor phenotype (2, 3). Diffuse gliomas are classified and graded according to histological criteria (oligodendroglioma, oligoastrocytoma, astrocytoma, and glioblastoma; grade II–IV). Glioblastoma is characterized by extensive tissue hypoxia, which is favorable for the induction and maintenance of a malignant phenotype (4). For glioma groups, tumor hypoxia is associated with anti-apoptosis, tumor recurrence, resistance to chemotherapy and radiation therapy, invasive potential, and decreased patient survival (5).

The importance of hypoxia in driving tumor immunosuppression and immune escape is receiving increased attention. Previous evidence indicates that T cells and natural killer (NK) cells in a hypoxic microenvironment exhibit an anergic or exhausted state, leading to dysfunction (6–8). Hypoxia promotes suppressive cells [regulatory T cells (Tregs) and tumor-associated macrophages (TAMs)] or immunosuppressive cytokines (e.g., TGFB1, IL-10, VEGFA, and ARG1), which in turn block immune effector cells (9). Currently, predictive biomarkers for immunotherapy mainly include programmed death-ligand 1 (PD-L1), microsatellite instability/defective mismatch repair (MSI/dMMR), and tumor mutational burden, but often ignore the problem of “poor soil” (10). Hence, tumor hypoxia may be exploited as a potential biomarker to predict immunotherapy outcomes.

At present, approaches to investigating tumor hypoxia are still limited. As comprehensive mRNA expression analysis has previously been used to identify potential biomarkers and reflects the current physiological state of the cell, this study used, for the first time, throughput mRNA profiling data to develop a hypoxia risk model as a prognostic biomarker to predict the immune microenvironment in glioma groups. In the future, this method may assist clinicians to make important treatment decisions.

## Materials and Methods

### Datasets

The RNA-seq transcriptome data and corresponding clinicopathological information of 575 LGG patients and 354 GBM patients were obtained from CGGA (www.cgga.org.cn) as a training set. Similarly, 530 LGG patients and 167 GBM patients from TCGA (http://cancergenome.nih.gov/) were downloaded as a validation set. The detail information was supplemented in Table S1. The RNA-seq transcriptome data were estimated as log2(x+1) transformed RSEM normalized counts. LGG and GBM datasets from TCGA were accessed by UCSC (https://xenabrowser.net/datapages/).

### Estimation of Immune Cell Type Fractions

CIBERSORT (https://cibersort.stanford.edu/) is an analytical tool developed by Newman et al. to provide an estimation of the abundances of member cell types in a mixed cell population, using gene expression data (11). In CIBERSORT, a leukocyte gene signature matrix consisting of 547 genes, which was termed LM22, was used to distinguish 22 immune cell types including naive B cells, memory B cells, plasma cells, CD8 T cells, naive CD4 T cells, resting memory CD4 T cells, activated memory CD4 T cells, follicular helper T cells, T cells regulatory (Tregs), gamma delta T cells, resting NK cells, activated NK cells, monocytes, macrophages M0, macrophages M1, macrophages M2, resting dendritic cells, activated dendritic cells, resting mast cells, activated mast cells, eosinophils, and neutrophils. We utilized CIBERSORT to estimate the fractions of 22 immune cell types between low and high hypoxia risk score.

### Constitution of a Risk Model

Hypoxia genes found to be statistically significant in univariable Cox regression were then used in multivariable Cox regression to achieve the coefficients; the risk-score formula was constructed as:

(1) $$\begin{array}{l} riskscore=\sum_{i=1}^{N}\left(Exp_{i}\times Coe_{i}\right) \end{array}$$

where *N* = 5, *Exp*_*i*_was the expression value of every five hypoxia genes, and the *Coe*_*i*_ was the corresponding multivariable Cox regression coefficient.

### Survival Analysis

OS was compared between the high and low hypoxia risk groups via Kaplan-Meier analysis using the survival and survminer packages in R. Univariate Cox analysis was performed to identify potential prognostic factors, and multivariate Cox analysis was used to determine risk score as an independent risk factor for OS in glioma. A ROC curve was generated to validate the accuracy of the risk model in predicting the patients' OS via the survivalROC R package.

### Gene Set Enrichment Analysis (GSEA)

GSEA was performed to detect a significant difference in the set of genes expressed between the high and low-risk groups in the enrichment of the MSigDB Collection (h.all.v7.0.cymbols.gmt; c5.bp.v7.0.symbols.gmt). Gene set permutations were performed 1,000 times for each analysis. The phenotype label was used as a risk score.

### Integration of Protein–Protein Interaction (PPI) Network

STRING database was utilized to develop a protein–protein interaction network (PPI). Cytoscape (https://cytoscape.org/) is an open source software platform for visualizing complex networks and integrating these with any type of attribute data (12). We used Cytoscape to construct a protein interaction relationship network and analyze the interaction relationship of the key genes in hypoxia associated genes. The Network Analyzer plug-in was then used to calculate node degree, defined as the number of interconnections to filter key genes of the PPI.

## Results

### Characterization of Hypoxia Risk Signature to Predict Glioma Prognosis

**Figure 1:**
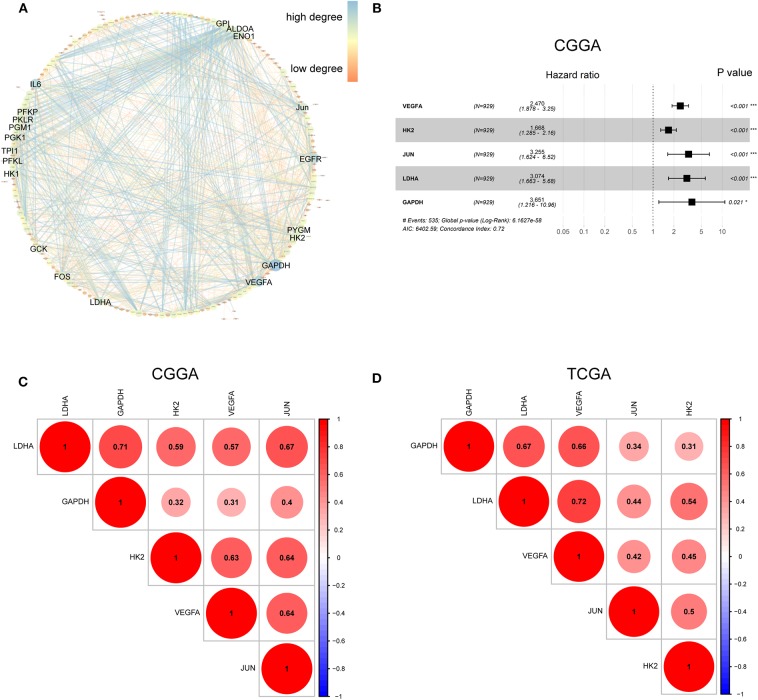
Characterization of hypoxia risk signature to predict prognosis of glioma. **(A)** Protein–Protein Interaction interactions among 200 hypoxia-associated genes. The 20 genes with the highest interaction degrees were labeled; **(B)** Construction of a hypoxia risk signature to predict glioma prognosis by univariate and multivariate Cox regression; **(C,D)** Spearman correlation analysis of five hypoxia genes in the CGGA and TCGA databases.

The hypoxia-related gene set was downloaded from Gene Set Enrichment Analysis (hallmark-hypoxia), which contained 200 genes upregulated in response to low oxygen levels. To better understand the interactions among these hypoxia-related genes, we conducted protein-protein interaction network analysis using the STRING online database (Available online: http://string-db.org) and Cytoscape software (Figure 1A). The 20 genes with the highest interaction degrees were identified, including GPI, ALDOA, ENO1, JUN, EGFR, PYGM, H2K, GAPDH, VEGFA, LDHA, FOS, GCK, HK1, PFKL, TPI1, PGK1, PGM1, PKLR, PFKP, and IL6, suggesting their important role in response to hypoxia.

To establish a hypoxia risk signature to predict glioma patients' prognoses, univariate and multivariate Cox regression analyses were performed using the top 20 genes in the CGGA training dataset. In the univariate Cox analysis, 20 hypoxia-related genes were significantly associated with patients' overall survival (OS). In the multivariate Cox analysis, five hypoxia-related genes with *P* < 0.05 were then chosen to build the predictive model consisting of VEGFA, HK2, JUN, LDHA, and GAPDH (Figure 1B). A risk-score formula was developed as following:

$$\begin{array}{l} \text{risk score}=\left(0.90\times \text{VEGFA}\right)+\left(0.51\times \text{HK}2\right)+\left(1.18\times \text{JUN}\right)\\ \;+\left(1.12\times \text{LDHA}\right)+\left(1.29\times \text{GAPDH}\right) \end{array}$$

All five genes were found to be significantly correlated with one another in both the CGGA (Figure 1C) and TCGA datasets (Figure 1D).

### Prognostic Value of the Hypoxia Risk Signature in Glioma Groups

**Figure 2:**
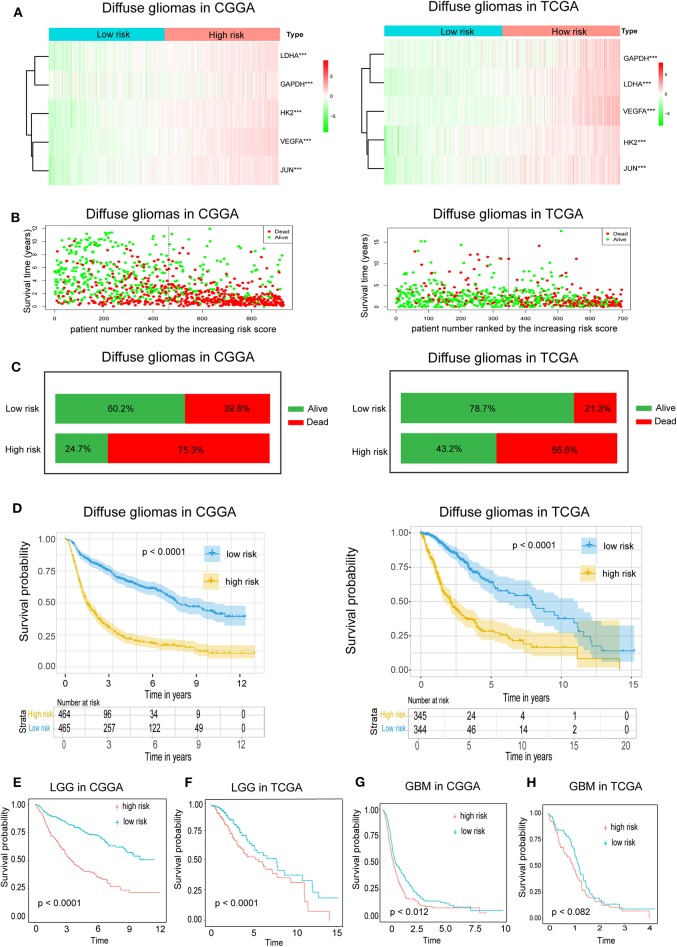
Prognostic value of the hypoxia risk signature in glioma. **(A)** A heatmap showing five hypoxia gene expression profiles in high and low hypoxia risk groups from the CGGA and TCGA databases; **(B)** Patient status distribution in the high and low hypoxia risk groups. The dot presents patient status ranked by the increasing risk score. The X axis is patient number and Y axis is survival time; **(C)** Mortality rates of the high and low hypoxia risk groups; **(D)** Kaplan-Meier overall survival curves for patients assigned to high and low hypoxia risk groups based on the risk score; **(E,F)** The prognosis values of hypoxia signature in LGG in the CGGA and TCGA data; **(G,H)** The prognosis values of hypoxia signature in GBM in the CGGA and TCGA data.

Because hypoxia often promotes a more aggressive tumor phenotype, we further investigated the prognostic value of the hypoxia signature. As shown in the heatmap (Figure 2A), the expressions of the five hypoxia-related genes were increased accompanying higher risk scores in both the CGGA and TCGA databases, implying that patients with high risk tend to develop a hypoxic microenvironment. Our data also showed that the mortality rate in the high-risk group was significantly higher than in the low-risk group (Figures 2B,C). Moreover, Kaplan-Meier analysis was performed to evaluate the prognostic value of the hypoxia signature in glioma. As shown in Figure 2C, high hypoxia risk score was associated with poor OS in the CGGA cohort, which was further validated by the TCGA cohort (Figure 2D).

We also determined the prognostic value of the hypoxia risk signature for different WHO grades. We found that patients with high risk scores had significantly shorter OS than those with low scores in LGG in the CGGA and TCGA cohorts (Figures 2E,F). In GBM, high risk predicted poor OS in the CGGA cohort, but not in the TCGA cohort (Figures 2G,H) (*p* = 0.07).

### Hypoxia Gene Expression Is Correlated With Clinicopathological Features in Gliomas

**Figure 3:**
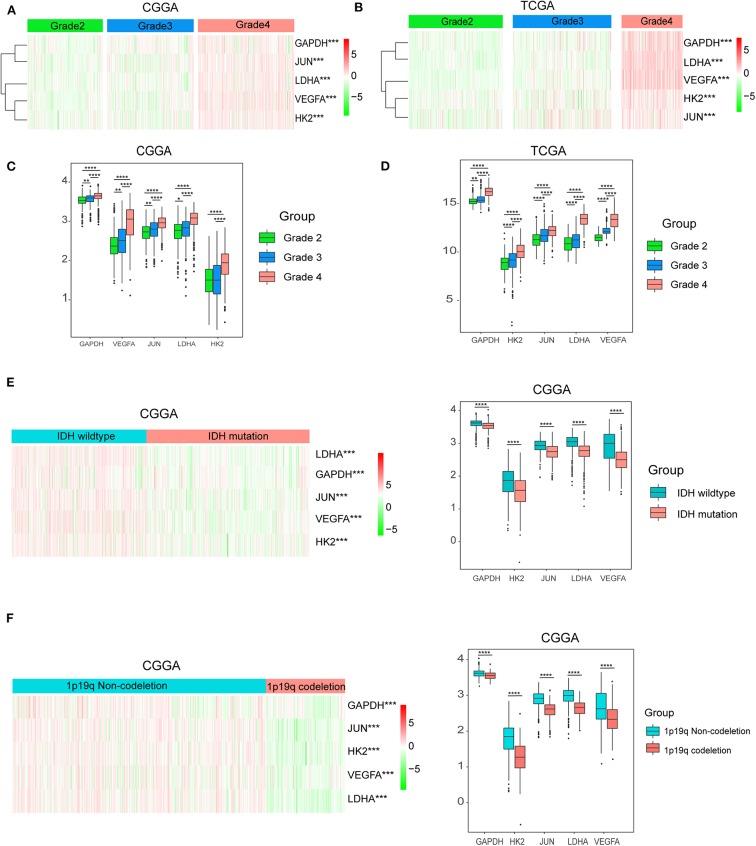
Hypoxia gene expression is correlated with clinicopathological features of gliomas. **(A,B)** Heatmaps showing five hypoxia gene expression profiles in different WHO grades from the CGGA and TCGA databases; **(C,D)** The expression levels of five hypoxia genes in gliomas with different WHO grades; **(E)** The expression levels of five hypoxia genes in gliomas with different IDH status; **(F)** The expression levels of five hypoxia genes in gliomas with different 1p/19q codeletion status; *P < 0.05, **P < 0.01, ***P < 0.001, and ****P < 0.0001.

Considering the important biological functions of hypoxia in tumorigenesis and development, we systematically investigated the relationships between the five identified hypoxia genes and the pathological features of gliomas, including WHO grade, IDH status, and 1p/19q codeletion status. Gene expression levels and WHO grades are presented as heatmaps (Figures 3A,B), showing that gene expression significantly increased in high WHO grades. The significant correlations between WHO grades and expression levels were also confirmed by quantitative analyses in both the CGGA (Figure 3C) and TCGA datasets (Figure 3D); as the WHO grade increased, the expression levels of the five genes were elevated.

We then studied the relationship of gene expression with 1p/19q codeletion status and IDH status, respectively. The results showed that expression levels of VEGFA, HK2, JUN, LDHA, and GAPDH were significantly high in glioma with wildtype-IDH (Figure 3E) and wildtype-1p/19q codeletion (Figure 3F).

### Hypoxia Risk Signature Shows Strong Power for Prognosis Assessment

**Figure 4:**
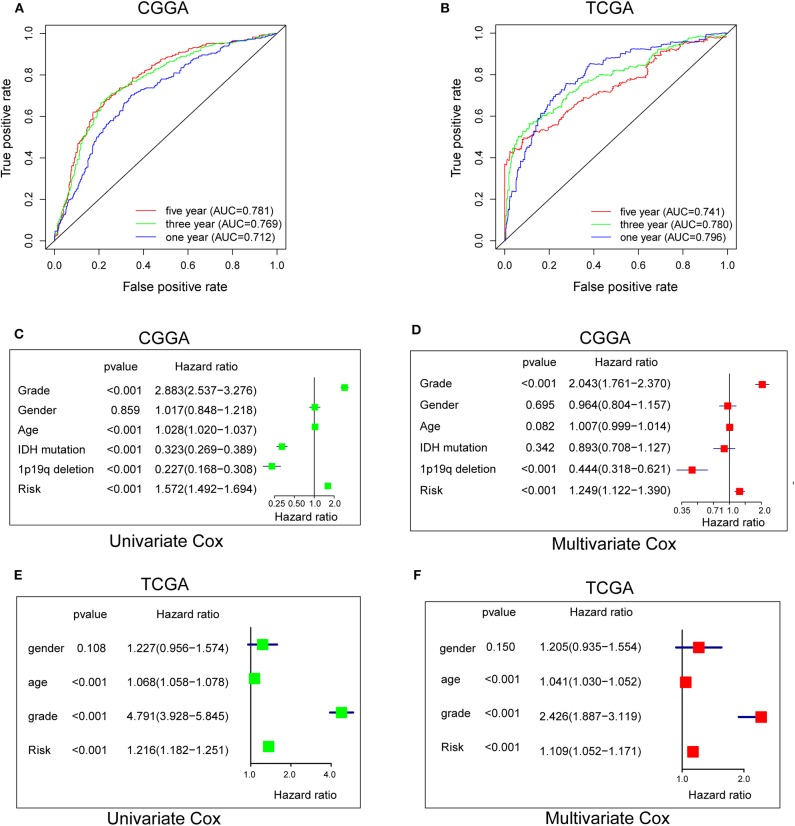
Prognostic value of the hypoxia risk signature in glioma. **(A,B)** ROC curves showing the predictive efficiency of the hypoxia risk signature on the 1-, 3-, and 5-years survival rate; **(C–F)** Univariate and multivariate Cox analyses evaluating the independent prognostic value of the hypoxia signature in terms of OS in glioma patients.

To evaluate the predictive efficiency of the hypoxia risk signature in the 1-, 3-, and 5-years survival rate, we performed a the received operating characteristic (ROC) curve utilizing the data from the CGGA and TCGA datasets. The area under the ROC curve (AUC) was 0.730 at 1-year, 0.776 at 3-years, and 0.798 at 5-years, respectively, indicating a high predictive value (Figure 4A). This was further validated by TCGA datasets (Figure 4B).

Patients with high risk score may develop hypoxia tumor microenvironment. The standard for high and low risk scores was evaluated on the basis of cut points associated with the Youden Index (derived from the AUROC for survival). Cut-off values of 14.4 for the risk model was identified, which served to divide the patients into high risk group (with levels of risk score ≥ 14.4) and a low risk group (with levels of risk score <14.4). Patients in high risk group (risk score ≥ 14.4) are associated with poor OS.

Univariate and multivariate Cox analyses were then applied to evaluate the independent prognostic value of hypoxia risk signature in terms of OS of glioma patients. The univariate analysis indicated that high hypoxia risk score was significantly correlated with poor OS (Figure 4C). Other variables related with poor survival included age, WHO grade, IDH status, and 1p19q status. Multivariate analysis showed that high hypoxia risk score was independently associated with significantly poorer OS of glioma patients (Figure 4D), which could serve as an independent prognostic factor for glioma. These were validated by the TCGA database (Figures 4E,F).

### GSEA Identifies Hypoxia-Related Signaling Pathways

**Figure 5:**
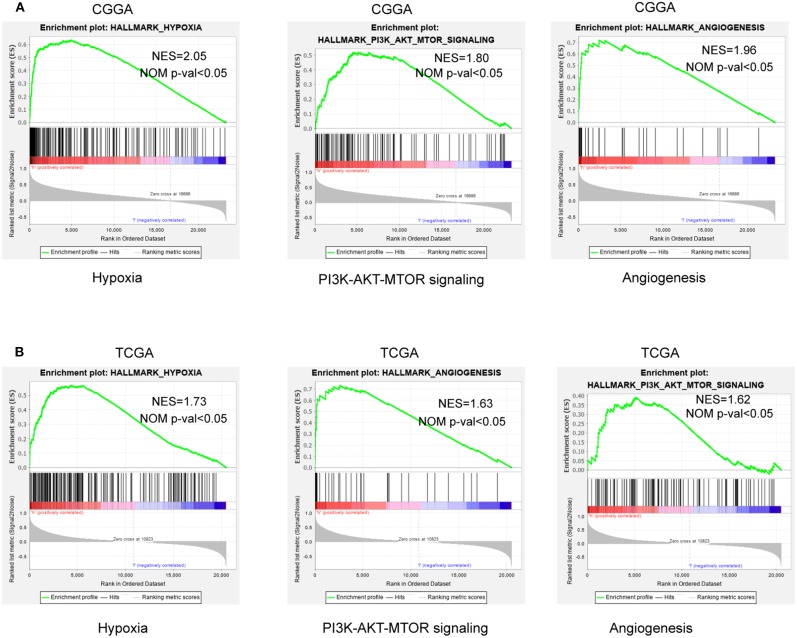
GSEA enrichment between low and high hypoxia risk groups. **(A)** GSEA revealing that genes in the high hypoxia risk group were enriched for hallmarks of malignant tumors in the CGGA data; **(B)** The results were further validated by the TCGA data. Normalized enrichment score (NES) > 1 and nominal *p*-value (NOM *p*-val) < 0.05 were considered significant gene sets.

To further verify associated signaling pathways activated in the high hypoxia risk group, we performed GSEA comparing the high and low hypoxia risk groups. Gene sets were differentially enriched in the high risk groups of the CGGA database, as they were related to processes that stimulate tumor proliferation and anti-apoptosis, such as hypoxia, DNA repair, PI3K-AKT-MTOR signaling, and angiogenesis (Figure 5A). These were further validated in the TCGA database (Figure 5B).

### Immune Landscape Between Low and High Hypoxia Risk Glioma Patients

Accumulating evidence suggests that a hypoxic microenvironment may protect tumors from natural anti-tumor immune responses by inhibiting anti-tumor immune effector cells and facilitating immune escape. Here, we investigated the capability of a hypoxia risk signature in evaluating the immune microenvironment.

**Figure 6:**
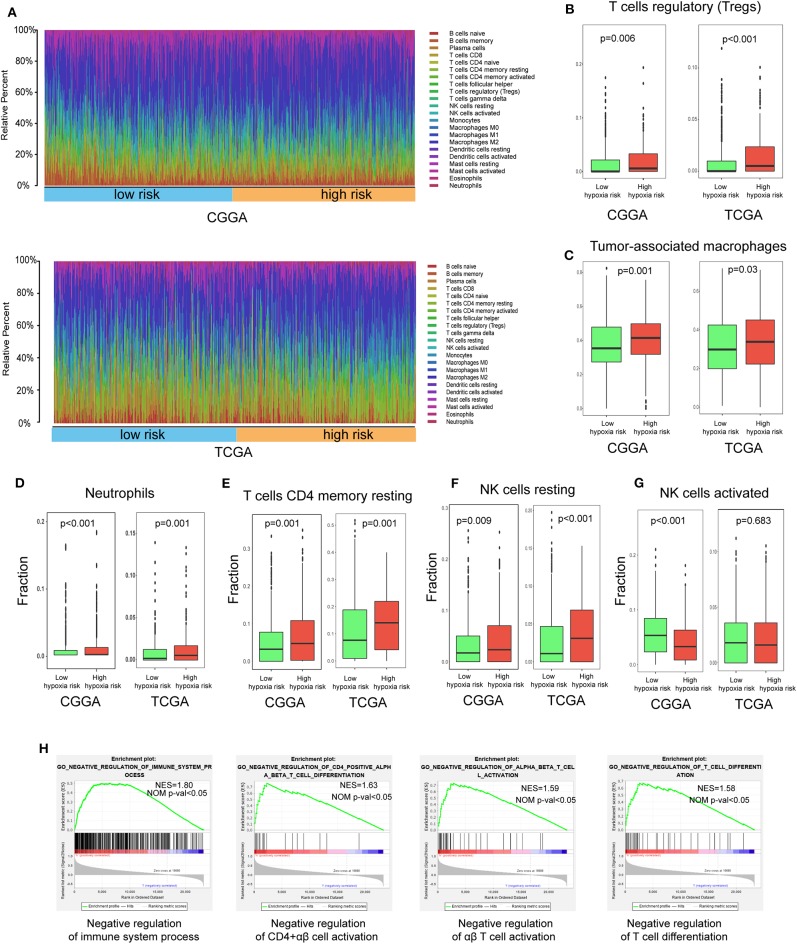
Immune landscape between low and high hypoxia risk glioma patients. **(A)** Relative proportion of immune infiltration in high and low hypoxia risk patients; **(B–G)** Box plots visualizing significantly different immune cells between high-risk and low hypoxia risk patients; **(H)** GSEA analysis revealing immune-related biological processes correlated with hypoxia signature.

Using the CIBERSORT method in combination with the LM22 signature matrix, we estimated differences in the immune infiltration of 22 immune cell types between low- and high-risk glioma patients. Figure 6A summarizes the results obtained from 929 glioma patients in CGGA and 697 patients in TCGA. Patients with high hypoxia risk had significantly higher proportions of immunosuppressive cells (e.g., Tregs, TAMs, and neutrophils) (Figures 6B–D), rested T cells, and NK cells (Figures 6E,F), but significantly lower proportions of activated NK cells (Figure 6G). Although there was no difference in the CD8^+^ T cells between high hypoxia risk tumors and low hypoxia risk tumors (data not shown), the immunosuppressive cells and inactivated NK cells may drive the immunosuppressive microenvironment.

Next, we analyzed immune-related biological processes correlated with the hypoxia gene signature. GSEA analysis was performed to analyze glioma samples with low or high risk. High risk gliomas were significantly enriched in negative regulation of the immunity pathway, such as negative regulation of B cells, CD4^+^ αβ cell activation, αβ T cell activation, and T cell differentiation (Figure 6H).

Therefore, targeting hypoxia may have significant clinical implications in improving immunotherapy.

### High Hypoxia Risk Score Indicates an Immunosuppressive Microenvironment

**Figure 7:**
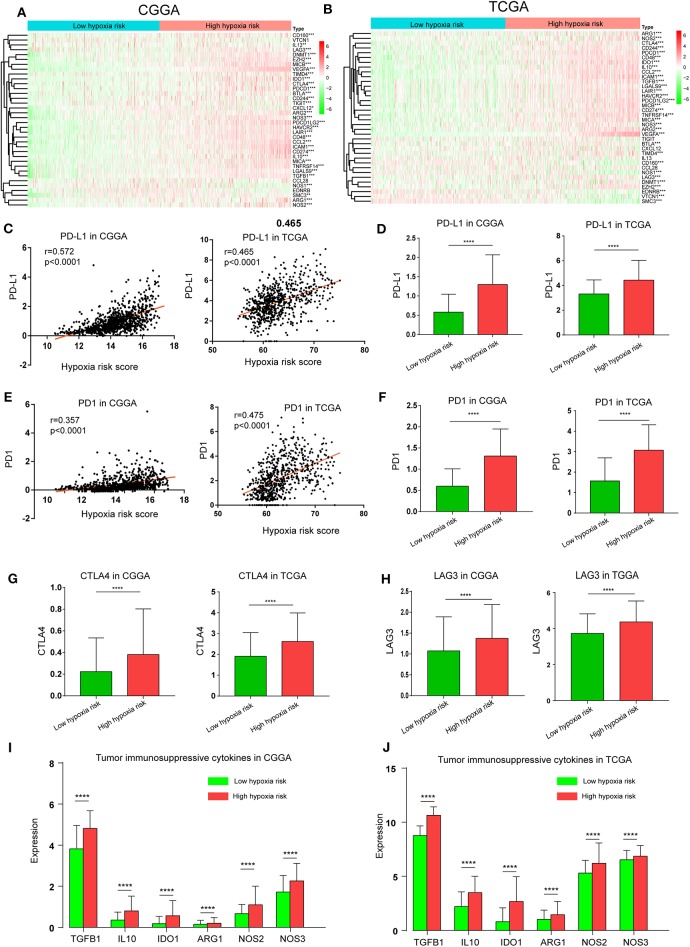
High hypoxia risk score indicates an immunosuppressive microenvironment. **(A,B)** Heatmap of the gene profiles involved in the negative regulation of the Cancer-Immunity Cycle in high and low hypoxia risk groups in the CGGA and TCGA databases; **(C)** Correlation between PD-L1 expression and hypoxia risk score; **(D)** PD-L1 expression in high and low hypoxia risk groups; **(E)** Correlation between PD1 expression and hypoxia risk score; **(F)** PD1 expression in high and low hypoxia risk groups; **(G)** CTLA-4 expression in high and low hypoxia risk groups; **(H)** LAG3 expression in high and low hypoxia risk groups; **(I,J)** Tumor immunosuppressive cytokine expression in high and low hypoxia risk groups. *P < 0.05, **P < 0.01, ***P < 0.001, and ****P < 0.0001.

The Cancer-Immunity Cycle has become the intellectual framework for cancer immunotherapy research. It describes a cycle of processes involving eradication of cancer by the immune system: cancer cell antigen release (step 1), cancer antigen presentation (step 2), priming and activation (step 3), trafficking of T cells to tumors (step 4), infiltration of T cells into tumors (step 5), recognition of cancer cells by T cells (step 6), and killing of cancer cells (step 7) (13). Here, we investigated the expression of genes negatively regulating these processes in low and high hypoxia risk groups. Genes signatures were download from Tracking Tumor Immunophenotype website (http://biocc.hrbmu.edu.cn/TIP/index.jsp) (14). As show in Figures 7A,B, genes involved in the negative regulation of the Cancer-Immunity Cycle were mostly upregulated in the high hypoxia risk group, indicating that patients in this group have low activities of the processes.

Based on previous evidence that immunosuppressive cytokines and immune checkpoints can be upregulated under hypoxic conditions, we investigated the expression of these molecules in the low and high hypoxia risk groups. Our results showed that PD1 and PD-L1, which positively correlated with hypoxia risk score, were upregulated in the high hypoxia risk group (Figures 7C–F). Additionally, the expression of critical immune checkpoints (i.e., CTLA-4 and LAG-3) in the high hypoxia risk group was significantly higher than that in the low group (Figures 7G,H). Immunosuppressive cytokines were also upregulated in the high hypoxia risk group (Figures 7I,J).

These results indicate that patients with high hypoxia risk scores tend to develop an immunosuppressive microenvironment via the upregulation of immunosuppressive cytokines and immune checkpoints.

### Prognostic Value of the Hypoxia Risk Signature in LGG

We further analyzed the prognostic value of the hypoxia risk signature in LGG. Our data also showed that the mortality rate in the high-risk group was significantly higher than in the low-risk group (Figures S1A,B). Moreover, ROC curve indicated a high predictive value of hypoxia risk signature in LGG (Figure S1C). Besides, univariate and multivariate Cox analyses showed that hypoxia risk signature could serve as an independent prognostic factor for LGG patients (Figures S1D,E).

### Prognostic Value of the Hypoxia Risk Signature in GBM

In GBM, patients in high-risk group have a higher mortality rate compared to low-risk group (Figures S2A,B). Besides, ROC curve showed a predictive value of hypoxia risk signature in GBM (Figure S2C), and univariate and multivariate Cox analyses revealed that hypoxia risk signature could serve as an independent prognostic factor for GBM patients (Figures S2D,E).

## Discussion

Substantial data suggest that tumor hypoxia is involved in processes conferring a growth advantage to tumor cells and the development of a more malignant phenotype (2, 3). Independent of standard prognostic factors, such as tumor stage, nodal status, tumor grade, and tumor hypoxia has been recommended as a prognostic factor for patient outcome (15). Although detecting the extent of hypoxia in patient tumors has been achieved using techniques, such as nitroimidazole, PET imaging, and biomarker expression by immunohistochemistry, a definitive approach is still unknown (16, 17).

The risk model created in this study consisted of five hypoxia-associated genes, most of which were upregulated under a hypoxic environment. It has been reported that VEGF, an important mitogen specific to endothelial cells, is dramatically induced by low oxygen tension in a variety of cell types and mediates hypoxia-induced angiogenesis (18). Similarly, HK2 and GAPDH are key mediators of aerobic glycolysis and promote tumor growth. Hypoxia inducible factor 1α (HIF1α) upregulates HK2 and GAPDH by binding to hypoxia-responsive element (HRE) promoters (19, 20). Various studies report that JUN and LDHA are also upregulated in hypoxic conditions (21–23).

In clinic, there are some available risk models based on multiple genes that can predict prognosis in patients with cancer. For example, the 21-gene expression assay (Oncotype DX, Genomic Health) is one of several commercially available gene-expression assays that provide prognostic information in hormone-receptor–positive breast cancer (24). National comprehensive cancer network (NCCN) clinical practice guidelines in breast cancer (Version 4.2018) has strongly recommended 21-gene expression assay for breast cancer patients. In our study, the risk model now consists of five genes representing a convenient detection in clinic.

Accumulating evidence suggests that a hypoxia may protect tumors from natural anti-tumor immune responses by (1) reducing activity of NK or CTL cells, (2) promoting suppressive cells (Tregs, TAMs, and neutrophils), and (3) increasing immunosuppressive molecular (25).

Activation of tumor antigen-specific T cells and NK cells is a critical event needed for anti-tumor immune surveillance. Many studies report that hypoxia inhibits T cell and NK cell growth and activation. For example, T cell growth and survival are impaired at low oxygen levels because hypoxia downregulates T cell IL-2 mRNA expression (26). Hypoxia could variably reduce NK cell ability to release IFNγ, GM-CSF, TNFα, CCL3, and CCL5 following PMA + ionomycin or IL15 + IL18 stimulation (27). Consistent with this evidence, our study demonstrated that for patients with high hypoxia risk, resting T cells and NK cells were increased while activated cells were decreased, indicating an immune disability status in this group.

Within the cancerous tissue, macrophages are designated as TAMs and classified into M1-like macrophage and M2-like macrophages. It is clear that M1-like macrophages can contribute to an antitumor response, and M2-like macrophages can promote angiogenesis, cell proliferation and immunosuppression. Tumor hypoxia is thought to play a vital role in the phenotypic control of TAMs, as hypoxic TAMs release factors that assist in tumor growth, cancer immunosuppression, and angiogenesis (28, 29). Previous studies support that the M2 phenotype of TAMs dominates in hypoxic niches (30). In our study, CIBERSORT showed that patients with high hypoxia risk had significantly higher proportions of M2 macrophages phenotype. Besides, immunosuppressive cells, like Treg cells and neutrophils, were increased in the high hypoxia group, indicating that our hypoxia risk model may predict the immune microenvironment.

Cytokines play an important role in regulating tumor immunity. Tumor immunosuppressive cytokines are one of the key factors contributing to immune cell exhaustion. In advanced malignancies, transforming growth factor-β (TGF-β) has been shown to suppress the immune system by inhibiting NK cell activity, decreasing cytokine production, inhibiting dendritic cell maturation, and altering T-cell cytotoxic properties (31). Interleukin-10 (IL-10), a key immune-suppressive cytokine secreted by M2-macrophages, Tregs and Th2-cells, has been shown to impair the proliferation, cytokine production, and migratory capacities of effector T cells (32). IL-10 has also contributed to sustained expression of Foxp3, TGFβ-Receptor-2, and TGF-β, thus stabilizing Treg phenotype and functions (33). In our study, immunosuppressive cytokines were upregulated in the high hypoxia risk group, which further promoted immunosuppression.

Immune checkpoints play a crucial role in carcinogenesis by promoting tumor immunosuppressive effects. Tumors can protect themselves from attack by stimulating immune checkpoint targets (e.g., PD1, PD-L1, LAG3, CTLA-4, TIGIT, and TIM-3). It is reported that hypoxia could induce a selective upregulation of PD-L1 on macrophages and MDSCs in the tumor microenvironment (34, 35). In our study, immune checkpoints like PD1, PD-L1, LAG3, and CTLA-4 were also upregulated in the high hypoxia risk group.

This study was the first of its kind to develop and validate a hypoxia risk model based on five hypoxia-associated genes; this model served as an independent prognostic factor for glioma patients and reflected the overall intensity of the immune response in the glioma microenvironment. Our research therefore offers a new understanding of how hypoxia status affects prognosis and the immune microenvironment in glioma and may benefit future hypoxia-targeted therapies for the tumor.

## Data Availability Statement

The datasets generated for this study can be found in the http://www.cgga.org.cn/, https://portal.gdc.cancer.gov/.

## Author Contributions

WL: conceptualization and writing. SW and XC: methodology. YY and YW: software. YP: validation. ZC: investigation. LC: data curation. XQ: formal analysis. SQ: project administration and funding acquisition. All authors have read and agreed to the published version of the manuscript.

## Conflict of Interest

The authors declare that the research was conducted in the absence of any commercial or financial relationships that could be construed as a potential conflict of interest.

## Supplementary Material

Figure S1 Prognostic value of the hypoxia risk signature in LGG. **(A)** LGG patient status distribution in the high and low hypoxia risk groups. The dot presents patient status ranked by the increasing risk score. The X axis is patient number and Y axis is survival time; **(B)** Mortality rates of the high and low hypoxia risk groups in LGG; **(C)** ROC curves showing the predictive efficiency of the hypoxia risk signature on the 1-, 3-, and 5-years survival rate in LGG; **(D,E)** Univariate and multivariate Cox analyses evaluating the independent prognostic value of the hypoxia signature in terms of OS in LGG patients.

Figure S2 Prognostic value of the hypoxia risk signature in GBM. **(A)** GBM patient status distribution in the high and low hypoxia risk groups. The dot presents patient status ranked by the increasing risk score. The X axis is patient number and Y axis is survival time; **(B)** Mortality rates of the high and low hypoxia risk groups in GBM; **(C)** ROC curves showing the predictive efficiency of the hypoxia risk signature on the 1-, 3-, and 5-years survival rate in GBM; **(D,E)** Univariate and multivariate Cox analyses evaluating the independent prognostic value of the hypoxia signature in terms of OS in GBM patients.

Table S1 Patient characteristics from CGGA and TCGA cohort.
