# Supplementary figures and images for: Characterization of Hypoxia Signature to Evaluate the Tumor Immune Microenvironment and Predict Prognosis in Glioma Groups
Source: Front Oncol. 2020 May 15;10:796. doi: 10.3389/fonc.2020.00796 (PMC7243125; doi:10.3389/fonc.2020.00796)

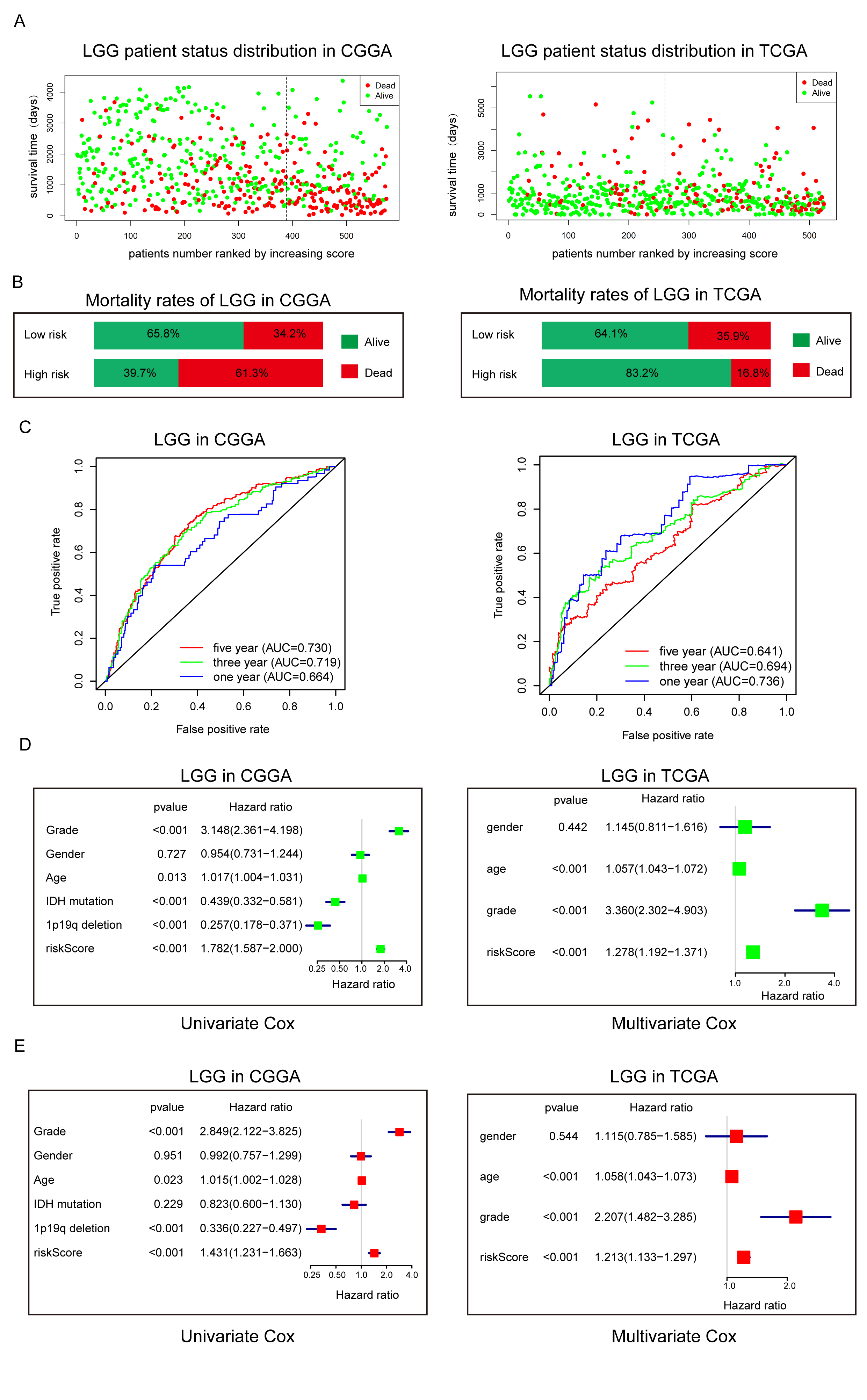

Supplement: Figure S1 — Prognostic value of the hypoxia risk signature in LGG. (A) LGG patient status distribution in the high and low hypoxia risk groups. The dot presents patient status ranked by the increasing risk score. The X axis is patient number and Y axis is survival time; (B) Mortality rates of the high and low hypoxia risk groups in LGG; (C) ROC curves showing the predictive efficiency of the hypoxia risk signature on the 1-, 3-, and 5-years survival rate in LGG; (D,E) Univariate and multivariate Cox analyses evaluating the independent prognostic value of the hypoxia signature in terms of OS in LGG patients. [file Image_1.TIF]

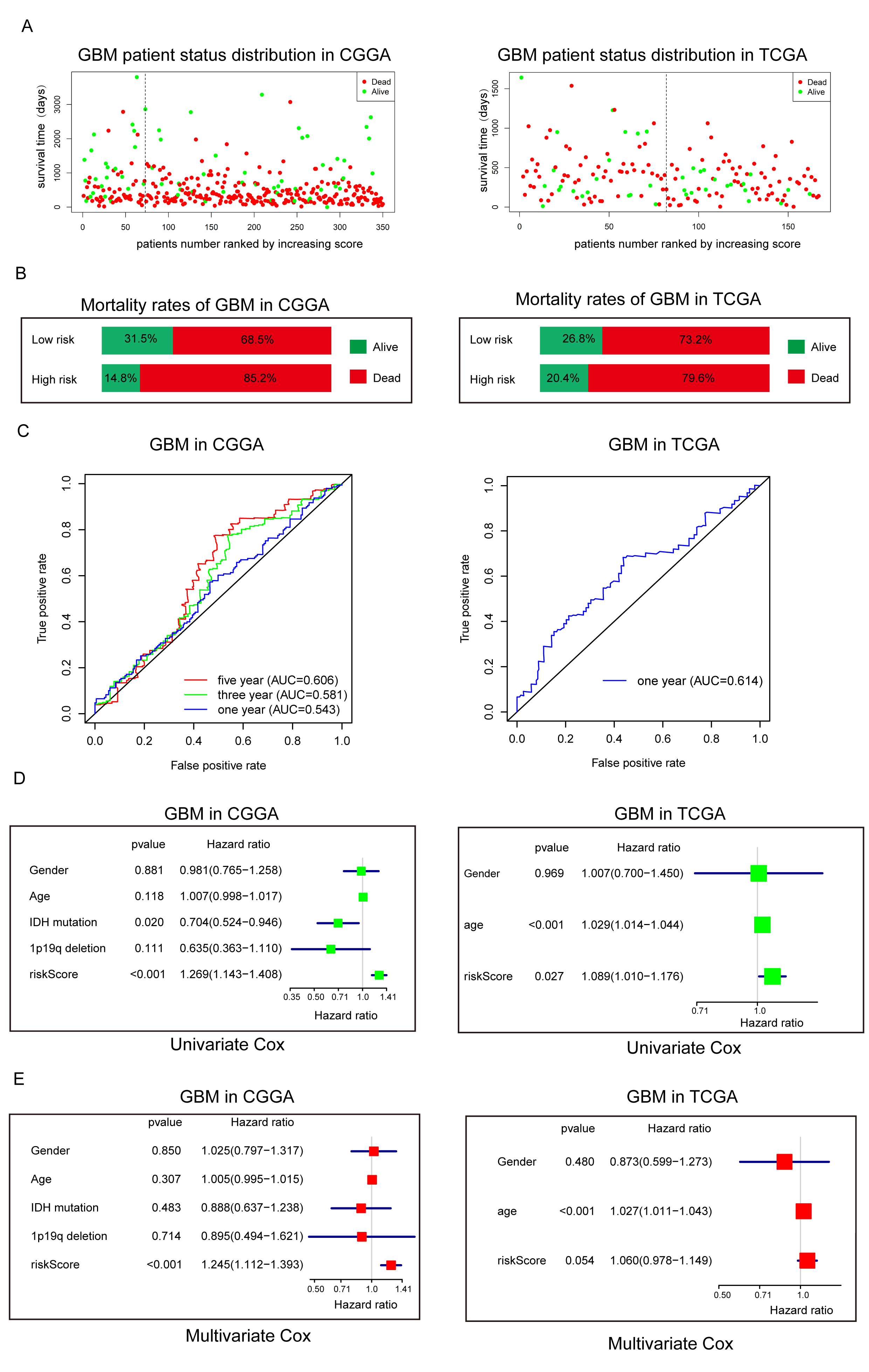

Supplement: Figure S2 — Prognostic value of the hypoxia risk signature in GBM. (A) GBM patient status distribution in the high and low hypoxia risk groups. The dot presents patient status ranked by the increasing risk score. The X axis is patient number and Y axis is survival time; (B) Mortality rates of the high and low hypoxia risk groups in GBM; (C) ROC curves showing the predictive efficiency of the hypoxia risk signature on the 1-, 3-, and 5-years survival rate in GBM; (D,E) Univariate and multivariate Cox analyses evaluating the independent prognostic value of the hypoxia signature in terms of OS in GBM patients. [file Image_2.TIF]
