# Supplementary material for: Characterization of Hypoxia Signature to Evaluate the Tumor Immune Microenvironment and Predict Prognosis in Glioma Groups
Source: Front Oncol. 2020 May 15;10:796. doi: 10.3389/fonc.2020.00796 (PMC7243125; doi:10.3389/fonc.2020.00796)
Supplement: Table S1 — Patient characteristics from CGGA and TCGA cohort. [file Table_1.DOCX]

**Supplementary table 1. Patient characteristics from CGGA and TCGA cohort.**

| Clinical characteristics | CGGA cohort | TGGA cohort |
| --- | --- | --- |
| Age at diagnosis (year)  ≥ 60  < 60  Gender  Male  Female  Grade  II  III  IV | 370  559  549  380  270  305  354 | 294  403  399  298  259  271  167 |
